# CD16 pre-ligation by defucosylated tumor-targeting mAb sensitizes human NK cells to γ_c_ cytokine stimulation via PI3K/mTOR axis

**DOI:** 10.1007/s00262-020-02482-2

**Published:** 2020-01-16

**Authors:** Cristina Capuano, Chiara Pighi, Roberta Maggio, Simone Battella, Stefania Morrone, Gabriella Palmieri, Angela Santoni, Christian Klein, Ricciarda Galandrini

**Affiliations:** 1 Department of Experimental Medicine, Sapienza University of Rome, Viale Regina Elena, 324, 00161 Rome, Italy; 2 Department of Molecular Medicine, Sapienza University of Rome, Rome, Italy; 3 Laboratorio Pasteur Italia Fondazione Cenci Bolognetti, Sapienza University of Rome, Rome, Italy; 4 IRCCS Neuromed, Pozzilli, Italy; 5 Roche Pharmaceutical Research and Early Development Roche Innovation Center Zurich, Schlieren, Switzerland; 6 Present Address: Clinical Cancer Research, Imperial College London, London, UK

**Keywords:** Obinutuzumab, CD16, Natural killer cells, IFN-γ, PI3K/mTOR, miR-155

## Abstract

Obinutuzumab is a glycoengineered tumor-targeting anti-CD20 mAb with a modified crystallizable fragment (Fc) domain designed to increase the affinity for the FcγRIIIA/CD16 receptor, which was recently approved for clinical use in CLL and follicular lymphoma. Here we extend our previous observation that, in human NK cells, the sustained CD16 ligation by obinutuzumab-opsonized targets leads to a markedly enhanced IFN-γ production upon a subsequent cytokine re-stimulation. The increased IFN-γ competence in response to IL-2 or IL-15 is attributable to post-transcriptional regulation, as it does not correlate with the upregulation of IFN-γ mRNA levels. Different from the reference molecule rituximab, we observe that the stimulation with obinutuzumab promotes the upregulation of microRNA (miR)-155 expression. A similar trend was also observed in NK cells from untreated CLL patients stimulated with obinutuzumab-opsonized autologous leukemia. miR-155 upregulation associates with reduced levels of SHIP-1 inositol phosphatase, which acts in constraining PI3K-dependent signals, by virtue of its ability to mediate phosphatidylinositol 3,4,5-trisphosphate (PIP3) de-phosphorylation. Downstream of PI3K, the phosphorylation status of mammalian target of rapamycin (mTOR) effector molecule, S6, results in amplified response to IL-2 or IL-15 stimulation in obinutuzumab-experienced cells. Importantly, NK cell treatment with the PI3K or mTOR inhibitors, idelalisib and rapamycin, respectively, prevents the enhanced cytokine responsiveness, thus, highlighting the relevance of the PI3K/mTOR axis in CD16-dependent priming. The enhanced IFN-γ competence may be envisaged to potentiate the immunoregulatory role of NK cells in a therapeutic setting.

**Electronic supplementary material:**

The online version of this article (10.1007/s00262-020-02482-2) contains supplementary material, which is available to authorized users.

## Introduction

NK cells doubly contribute to the therapeutic efficacy of tumor-targeting mAbs; besides the killing of tumor cells mediated by the engagement of the low affinity receptor for IgG, FcγRIIIA/CD16, activated NK cells secrete pro-inflammatory cytokines and chemokines which act in boosting the recruitment and activation of other immune effector cells and the development of long-lasting T cell immunity [2, 3]. In particular, NK-derived IFN-γ stands as a key immunoregulatory factor in the shaping of anti-tumor adaptive immune responses, by promoting the maturation of DC and the subsequent development of Th1 and CTL responses [4]. Indeed, the current understanding of anti-tumor responses driven by tumor-targeting mAbs introduced a novel ground by which NK cells may contribute to the development of a vaccine-like effect required for the long-term protection of mAb-treated patients [5–9].

By virtue of an accessible *Ifng* locus, NK cells represent a prompt source of IFN-γ. Such cytokine is transcribed constitutively at low levels in NK cells; its increased production in response to cytokines or after the engagement of activating receptors is tightly regulated at transcriptional and post-transcriptional levels [10–12]. In this context, microRNA (miR)-155 functions as a positive regulator of IFN-γ production stimulated by CD16 and cytokines [13] by directly targeting the hematopoietic cell-specific inositol 5-phosphatase, SHIP-1, which negatively regulates the PI3K pathway [14]. Downstream PI3K, the master metabolic regulator mammalian target of rapamycin (mTOR) promotes IFN-γ translation through the phosphorylation of the ribosomal protein S6 kinase (S6K) and the eukaryotic translation initiation factor 4E (eIF4E)-binding protein 1 (4E-BP1) [15–18].

To reach an enhanced clinical efficacy, new mAbs with increased affinity for CD16 have been generated. Among them, obinutuzumab, recently approved for clinical use [19–21], is a type II glycoengineered anti-CD20 mAb with a defucosylated crystallizable fragment (Fc) domain that binds to a CD20 epitope in a different space orientation with respect to the reference molecule rituximab [22, 23].

Our recent studies have revealed that the strength of CD16 ligation by tumor-targeting mAbs impacts on receptor signaling and functional properties [24–26].

Here, extending our previous observations [25], we demonstrate that following obinutuzumab pre-stimulation, NK cells undergo enhanced IFN-γ production in response to a subsequent re-stimulation with common γ chain (γ_c_) cytokines IL-15 or IL-2, which correlates to the upregulation of miR-155 and to reduced SHIP-1 levels but not with the upregulation of IFN-γ mRNA levels; the increased IFN-γ competence depends on the PI3K/mTOR axis.

Such data add mechanistic insights into NK cell plasticity in therapeutic settings. Moreover, taking into account the current research efforts focused on the development of IL-2 and IL-15 cytokine variants with extended half-life and targeted action [27], our results suggest that obinutuzumab-based immunotherapy in combination with NK cell-activating cytokines may achieve a useful synergism for the development of long-lasting curative anti-tumor responses.

## Materials and methods

### Antibodies

The following anti-CD20 mAbs were used: the chimeric IgG1κ rituximab, the humanized IgG1κ obinutuzumab (GA101), and its non-glycoengineered parental molecule, GA101 wild type (WT), all kindly provided by Roche Innovation Center Zurich (Schlieren, Switzerland).

For functional assays, the following mAbs were used: anti-2B4 (clone:C1.7, #IM1607, Beckman Coulter Life Science), anti-NKp46 (clone: 9E2, #331902, Biolegend), anti-natural killer group 2 member D (NKG2D) (clone: 149810, #MAB139, R&D Systems), all mouse IgG1 isotype, and goat F(ab')_2_ fragment anti-mouse IgG (H + L) (#115-006-003, Jackson ImmunoResearch Laboratories). The following fluorochrome-conjugated mAbs were used for flow cytometric analysis: anti-CD25 APC (clone:M-A2511, #555434) and anti-CD215 PE (clone:JM7A4, #566589) were from BD Biosciences; the anti-pS6 ribosomal protein (S235/236) PE (clone: D57.2.2E, #5316S) was from Cell Signaling Technology. For immunoblot analysis, antibodies were obtained from the following sources: anti-SHIP-1 (clone:P1C1, #sc-8425) from Santa Cruz Biotechnology Inc); the anti-phospho-STAT4 (Tyr693) (clone:D2E4, #4134), anti-STAT4 (clone:C46B10, #2653), anti-Src homology 2 domain-containing leukocyte protein of 76 kDa (SLP-76) (#4958) and anti-Akt (#9272), all from Cell Signaling Technology.

### Patients and healthy donors

PBMCs were obtained from anonymized healthy donors of Transfusion Center of Sapienza University (Rome, Italy) or CLL patients of Hematology Unit, S. Maria Goretti Hospital (Latina, Italy).

The diagnosis of CLL was based on criteria recommended by the International Workshop on Chronic Lymphocytic Leukemia (IWCLL); the stage of disease was assessed according to the Rai staging system [24]. In all specimens, the percentage of CD5^+^CD19^+^ CLL cells was more than 70%. From each patient, part of PBMC was used to obtain primary cultured NK cells (see below) and the rest was cryopreserved and stored at − 160 °C. The day before the experiment, thawed samples were rested overnight in complete medium. Only samples with viability more than 90% were used.

### Cell system

Primary cultured human NK cells were obtained from healthy donors [28], or from CLL patients as previously described [24]. Briefly, PBMC were co-cultured for 10 days with irradiated (3000 rad) Epstein–Barr virus positive RPMI 8866 lymphoblastoid cell line in 10% FCS and 1% l-glutamine containing RPMI 1640 (all from Euroclone). Experiments were performed on NK cell cultures which were more than 80% pure (CD3^−^CD56^+^).

### Anti-CD20-experienced NK cell preparation and purification

The human CD20^+^ lymphoblastoid Raji cell line or primary B-CLL cells were loaded with 10 μγ/ml of EZ-Link Sulfo-NHS-SS-Biotin (#21331, Thermo Fisher Scientific) for 30 min at room temperature [29], washed twice with PBS (Euroclone) and then opsonized for 20 min at room temperature with saturating doses of rituximab, obinutuzumab or obinutuzumab WT. Not opsonized and anti-CD20-opsonized targets were mixed at 1:2 ratio with primary cultured NK cells for 18 h, and then washed twice with cold 5 mM EDTA containing PBS. NK cells were purified by negative selection on biotin-binder Dynabeads (#11047), followed by anti-CD3-coated Dynabeads (#11151D) (both from Invitrogen, Life Technologies), according to the manufacturer’s protocols. Experienced NK populations were checked by flow cytometry to assess purity.

### Evaluation of IFN-γ release

Purified experienced NK cells (5 × 10^5^/ml) were resuspended in complete medium and left untreated or stimulated for 18 h at 37 °C with 500 U/ml of IL-2 (#200-02), or 10 ng/ml of IL-12 (#200-12), or 100 ng/ml of IL-15 (#200-15) (all from PeproTech). Where required, 10 μM idelalisib (CAL-101, GS1101; #S2226, Selleck Chemicals) or 20 nM rapamycin (#R0395, Merck) was added 2 h before cytokine treatment, and maintained until the end of stimulation. An equivalent volume of DMSO (#D5879, Merck), as vehicle, was added to control samples.

Stimulation of activating receptors was performed by plastic-immobilized goat *F*(*ab*ʹ)_2_ anti-mouse IgG (H + L) (1 μg/10^6^) followed by anti-NKp46 (0.5 μg/10^6^), anti-NKG2D (1 μg/10^6^) or anti-2B4 (0.24 μg/10^6^) mAbs, used alone or in combination. IFN-γ was quantified in the supernatants by commercial ELISA kit (#EHIFNG2, Thermo Fisher Scientific), according to the manufacturer’s instructions.

### Stimulation and analysis of S6 phosphorylation

To determine the phosphorylation status of ribosomal protein S6, purified experienced NK cells were resuspended in complete medium, rested for 2 h at 37 °C and stimulated with 500 U/ml of IL-2 or 100 ng/ml of IL-15 at 37 °C for different lengths of time. Following stimulation, samples were washed, fixed and permeabilized with commercial kit (# 00-5523-00, eBioscience, Thermo Fisher Scientific), according to the manufacturer’s instructions, and stained with PE-conjugated anti-pS6 (S235/236) rabbit mAb. Samples were acquired on a FACSCanto II (BD Bioscience) and analyzed with FlowJo v.9.3.2 (TreeStar) software.

### miR and mRNA analyses

Total RNA, including miRs, was extracted from highly purified experienced NK cells before and after cytokine stimulation using Total RNA Purification Kit (#3755, Norgen Biotek Corp). The concentration and purity of extracted RNA were determined spectrophotometrically using NanoDrop 2000 (Thermo Fisher Scientific). cDNA was generated by means of High-Capacity cDNA Reverse Transcription kit (#4374966) or TaqMan MicroRNA Reverse Transcription kit (#4366596), using RT primers specific for miR-155, miR-29a, RNU-44 and RNU-48. mRNA, and miR levels were determined by real-time quantitative PCR (RT-qPCR) using TaqMan gene expression or miR assays, according to the manufacturer’s instructions. All reagents were obtained from Applied Biosystem, Life Technologies (Thermo Fisher Scientific) and the list of all primers employed is reported below. Reactions were performed in 96-well plate format by StepOnePlus real-time PCR system and data obtained were analyzed by StepOne Software v2.3 (Applied Biosystem). Water (no template) was loaded as a negative control. mRNA and miR contents were normalized to glyceraldehyde-3-phosphate dehydrogenase (GAPDH) or RNU-44 and RNU-48 endogenous controls, respectively, and relative quantification was evaluated by the comparative cycling threshold (ΔΔC_T_) method. The fold change was calculated according to the formula 2^−ΔΔ*C*T^, setting the control population to 1. The following TaqMan gene expression assays (#4331182) were used: Hs00989291_m1 IFNG, Hs00183290_m1 INPP5D, Hs00705164_s1 suppressor of cytokine signalling 1 (SOCS-1), Hs03929097_g1 GAPDH. The following TaqMan miRNA assays (#4427975) were used: hsa-miR-155 (#002623), hsa-miR-29a-5p (#002447), RNU-44 (#001094), RNU-48 (#001006) all conjugated with fluorochrome carboxyfluorescein (FAM).

### Biochemical analysis

For assessing STAT4 phosphorylation, purified experienced NK populations were stimulated for 30 min at 37 °C with 500 U/ml of IL-2 or 100 ng/ml of IL-12, or left untreated, and lysed in radio-immunoprecipitation assay (RIPA) buffer (50 mM Tris–HCl, pH 7.5, 150 mM NaCl, 0.5% deoxycholate, 0.1% SDS, 1 mM EDTA, pH8, 1 mM ethylene glycol tetraacetic acid (EGTA), pH 8, 5 mM MgCl_2_, 5 mM NaF) supplemented with 1 mM phenylmethylsulfonyl fluoride, 1 mM Na_3_VO_4_, 1 μg/ml each of aprotinin and leupeptin inhibitors. To analyze SHIP-1, SLP-76 and Akt levels, whole cell lysates of purified experienced NK cells were obtained by incubating with 1% Triton X-100 lysis buffer (50 mM Tris–HCl, pH 7.5, 150 mM NaCl, 1 mM EGTA, pH 8, 1 mM MgCl_2_, 50 mM NaF) supplemented with protease and phosphatase inhibitors as above. Protein content was determined by Bradford Colorimetric Assay (#500-0006, Bio-Rad Laboratories) and equal amounts of proteins from each sample were separated by SDS-PAGE and transferred to nitrocellulose for immunoblot analysis. Quantification of specific bands was performed with ImageJ1.41o software (National Institutes of Health).

### Statistical analysis

Wilcoxon matched-pairs signed rank or paired *t* tests were used to determine statistically significative differences (*p* value < 0.05) between two groups, as appropriate. Error bars represent the SEM. Analysis were performed using Prism v.6 (GraphPad Software).

## Results

### Pre-stimulation of CD16 with obinutuzumab-opsonized targets leads to an amplified IFN-γ production in response to IL-2 or IL-15, independently of transcriptional regulation

**Fig. 1:**
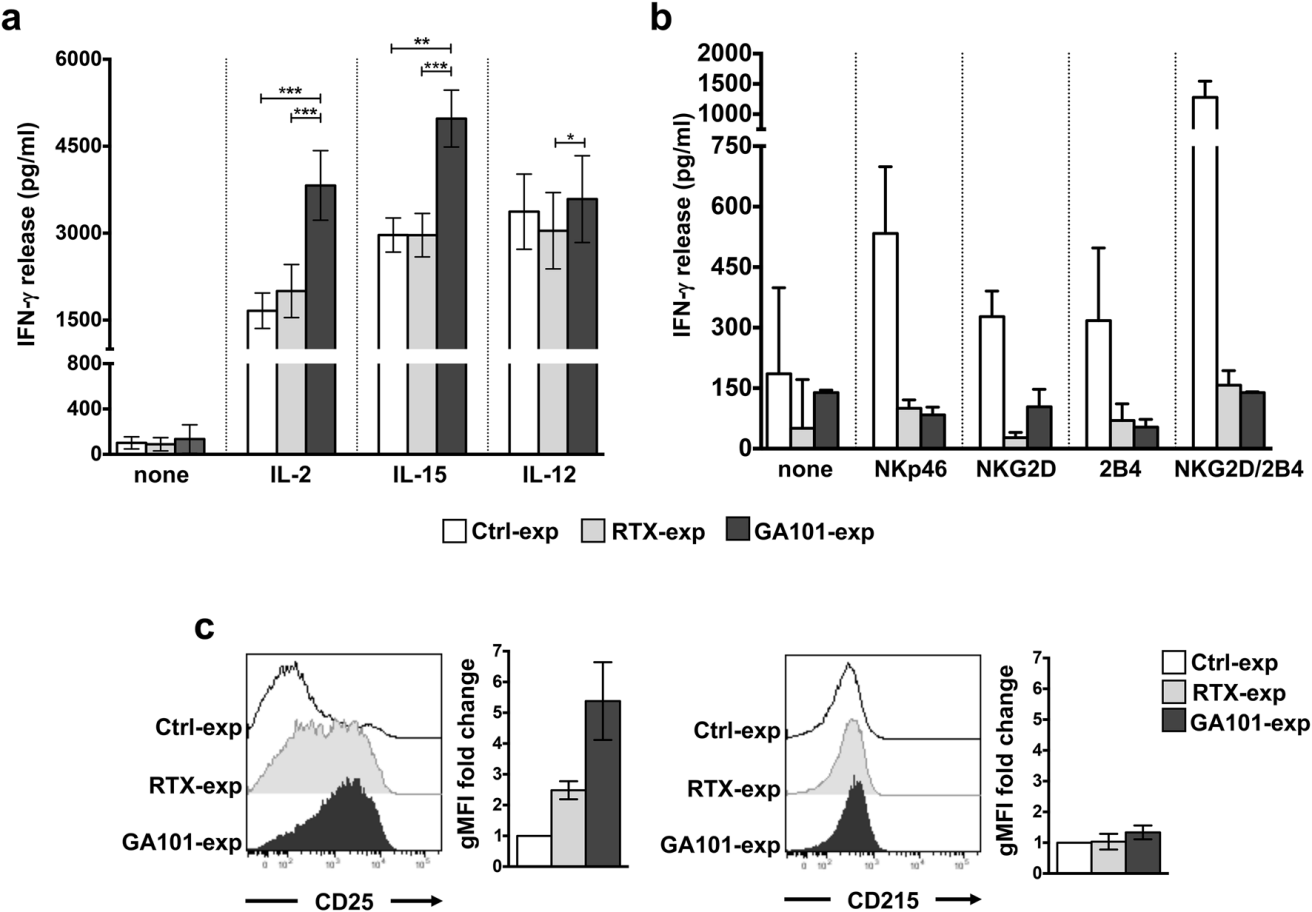
Obinutuzumab-mediated CD16 pre-ligation induces an enhanced IFN-γ production in response to IL-2 or IL-15 stimulation. Primary cultured NK cells were immunomagnetically purified by negative selection upon 18 h of co-culture (2:1) with biotinylated rituximab (RTX-exp)-, obinutuzumab (GA101-exp)-opsonized or not opsonized Raji (Ctrl-exp). Experienced cells were left untreated (none) (**a**, **b**) or stimulated with (**a**) IL-2 (500 U/ml), IL-15 (100 ng/ml) or IL-12 (10 ng/ml) or with (**b**) the indicated plastic-immobilized mAbs. After 18 h, IFN-γ levels were measured in the collected supernatants. **a** Data are presented as mean ± SEM of *n* = 14 donors for IL-2 setting (***p = 0.0001), *n* = 11 donors for IL-15 setting (***p = 0.001, **p = 0.002) or *n* = 7 donors for IL-12 setting (*p = 0.0156) in five independent experiments. **b** Mean ± SEM from *n* = 3 donors are reported. **c** IL-2R α (CD25) or IL-15R α (CD215) expression was assessed by FACS analysis in purified experienced NK cells. Histogram overlays from one representative experiment are shown**.** Geometric MFI (gMFI) expressed as fold change with respect to Ctrl-exp population (set to 1) is reported. Data (mean ± SEM) from *n* = 3 donors are reported

In line with our recent data demonstrating that the sustained CD16 pre-ligation in high-affinity conditions primes NK cells for IFN-γ production [25], we show here that primary cultured NK cells that were stimulated for 18 h with obinutuzumab-opsonized Raji cells (obinutuzumab-experienced NK cells) became able to secrete an increased amount of IFN-γ in response to high doses of IL-2 (500 U/ml) or IL-15 (100 ng/ml) and, to a lesser extent, of IL-12 (10 ng/ml), with respect to NK cells that were stimulated with rituximab-opsonized (rituximab-experienced NK cells) or not opsonized (control population) Raji cells, which behave comparably (Fig. 1a). The enhanced IFN-γ production was selectively observed in response to cytokine stimulation; indeed, we found a marked impairment of the ability to secrete IFN-γ in response to either ITAM-dependent (i.e., NKp46) or -independent (i.e., NKG2D and 2B4) receptors, alone or in combination, both in rituximab- and in obinutuzumab-experienced cells, with respect to control population (Fig. 1b).

To assess whether the increased IFN-γ production may be attributable to the modulation of specific cytokine receptor subunits, we evaluated IL-2 receptor (IL-2R) α-chain (CD25) and IL-15R α-chain (CD215) expression levels. While a robust upregulation of IL-2R α surface levels was observed in obinutuzumab- and, to a lesser extent, in rituximab-experienced cells, IL-15R α levels remained unaffected (Fig. 1c), thus indicating that the increased cytokine responsiveness does not completely rely on receptor affinity modulation.

**Fig. 2:**
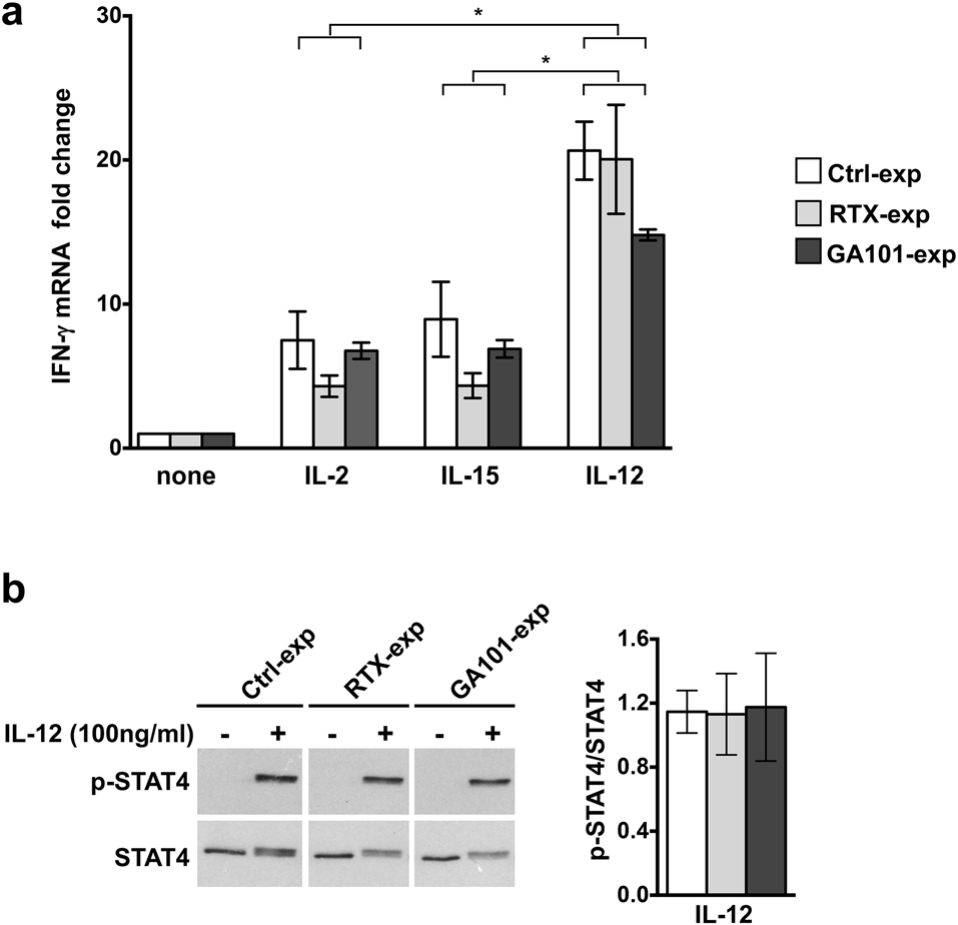
Cytokine-induced IFN-γ mRNA transcription in anti-CD20-experienced cells. **a** Purified experienced NK cells were obtained and stimulated with cytokines as in Fig. 1a. After 18 h, total RNA was extracted from untreated (none) or cytokine-activated NK cells and IFN-γ mRNA levels were measured by RT-qPCR. The fold change expression relative to untreated populations (set to 1) after normalizing with the GAPDH endogenous control is reported in the bar graph. Data (mean ± SEM) from *n* = 6 donors of three independent experiments are shown, *p = 0.0313. **b** Purified experienced NK cells were stimulated as indicated for 30 min. Western blot analysis of equal amounts of total lysates (4 × 10^5^cells/point) were probed with anti-phospho-STAT4 (p-STAT4) followed by anti-STAT4 mAbs. All lanes were from the same experiment but were not contiguous. One representative experiment out of three performed is shown (left panels). Densitometric analysis of phosphorylated STAT4 (p-STAT4) normalized to total STAT4 in IL-12-stimulated samples is represented. Data (means ± SEM) from *n* = 3 donors (right panel)

To address the impact of cytokine stimulation on IFN-γ transcription, we analyzed IFN-γ mRNA levels. We observed that mRNA levels induced by IL-2 or IL-15 stimulation are comparable among all the experimental groups, indicating that the increased IFN-γ production in obinutuzumab-experienced cells is independent of transcriptional regulation. By contrast, IL-12 stimulation induces higher levels of IFN-γ transcript, in spite of comparable levels of IFN-γ release (Fig. 2a). In line with the necessary and sufficient role of STAT-4 phosphorylation for IL-12-induced IFN-γ transcription, we observed that IL-12 stimulation efficiently induces STAT-4 phosphorylation at comparable levels between all the experimental groups (Fig. 2b).

Overall, these data demonstrate that in obinutuzumab-experienced cells the enhanced IFN-γ production in response to γ_c_ cytokine stimulation relies on post-transcriptional events.

### miR-155 upregulation in obinutuzumab-experienced NK cells is associated with reduced SHIP-1 levels

We analyzed the levels of selected miRs previously involved in the regulation of IFN-γ production in NK cells [30, 31]. It is known that both miR-155 and miR-29a are poorly expressed in primary human NK cells and that their increased levels in activated cells act in the regulation of IFN-γ production [13, 32–34].

**Fig. 3:**
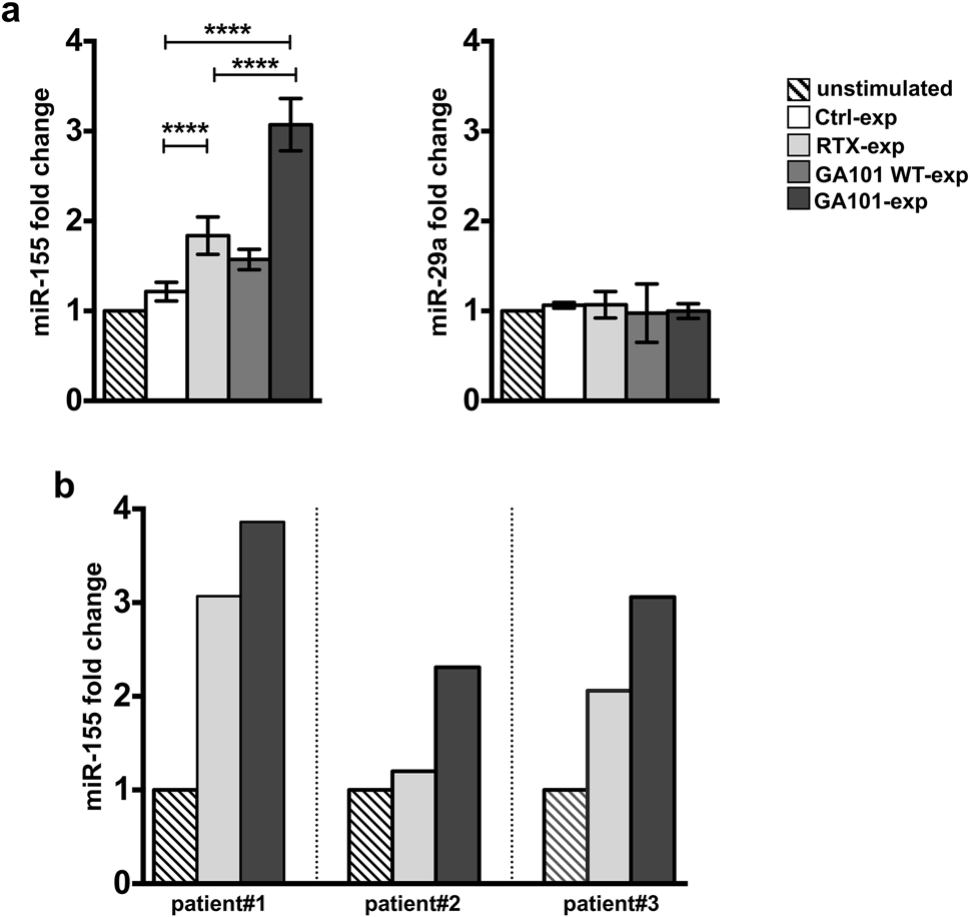
Obinutuzumab-mediated CD16 ligation upregulates miR-155 levels in NK cells from healthy donors and CLL patients. **a** Primary cultured NK cells were stimulated (2:1) for 18 h with biotinylated rituximab (RTX-exp)-, obinutuzumab WT (GA101 WT-exp)-, obinutuzumab (GA101-exp)-opsonized, not opsonized Raji (Ctrl-exp) or left unstimulated and immunomagnetically purified by negative selection, or **b** NK cells from three untreated CLL patients were stimulated with biotinylated rituximab (RTX-exp)-, obinutuzumab (GA101-exp)-opsonized autologous leukemia or left unstimulated and purified as in **a**. Relative miR-155 (**a**, **b)** or miR-29a (**a**) levels were measured by RT-qPCR. Bar graphs depict the fold change expression relative to the unstimulated population (set to 1) after normalizing with the RNU-44 endogenous control. **a** Data (mean ± SEM) from *n* = 17 donors from five independent experiments are shown. ****p < 0.0001

We quantified miR-155 and miR-29a by RT-qPCR by normalizing with RNU-44 endogenous control. Our data show that, upon 18 h of interaction with obinutuzumab-opsonized cells, miR-155 levels undergo significant upregulation, with respect to rituximab-stimulated cells or control population, which behaves comparably to unstimulated NK cells. Although at lower degree, but still significant with respect to control population, the upregulation of miR-155 was detected in rituximab-experienced cells. The observation that the fucosylated WT version of obinutuzumab, GA101WT, behaves similarly to rituximab, indicates that the strength of CD16 stimulation affects the levels of miR-155. On the contrary, no major modulation of miR-29a was observed (Fig. 3a).

Remarkably, we found higher miR-155 levels in obinutuzumab- compared to rituximab-experienced NK cells also in primary NK cells derived from untreated CLL patients (Supplementary Table 1) stimulated for 18 h with anti-CD20-opsonized autologous leukemia (Fig. 3b). Similar results were obtained using RNU-48 as endogenous control (Supplementary Fig. 1).

**Fig. 4:**
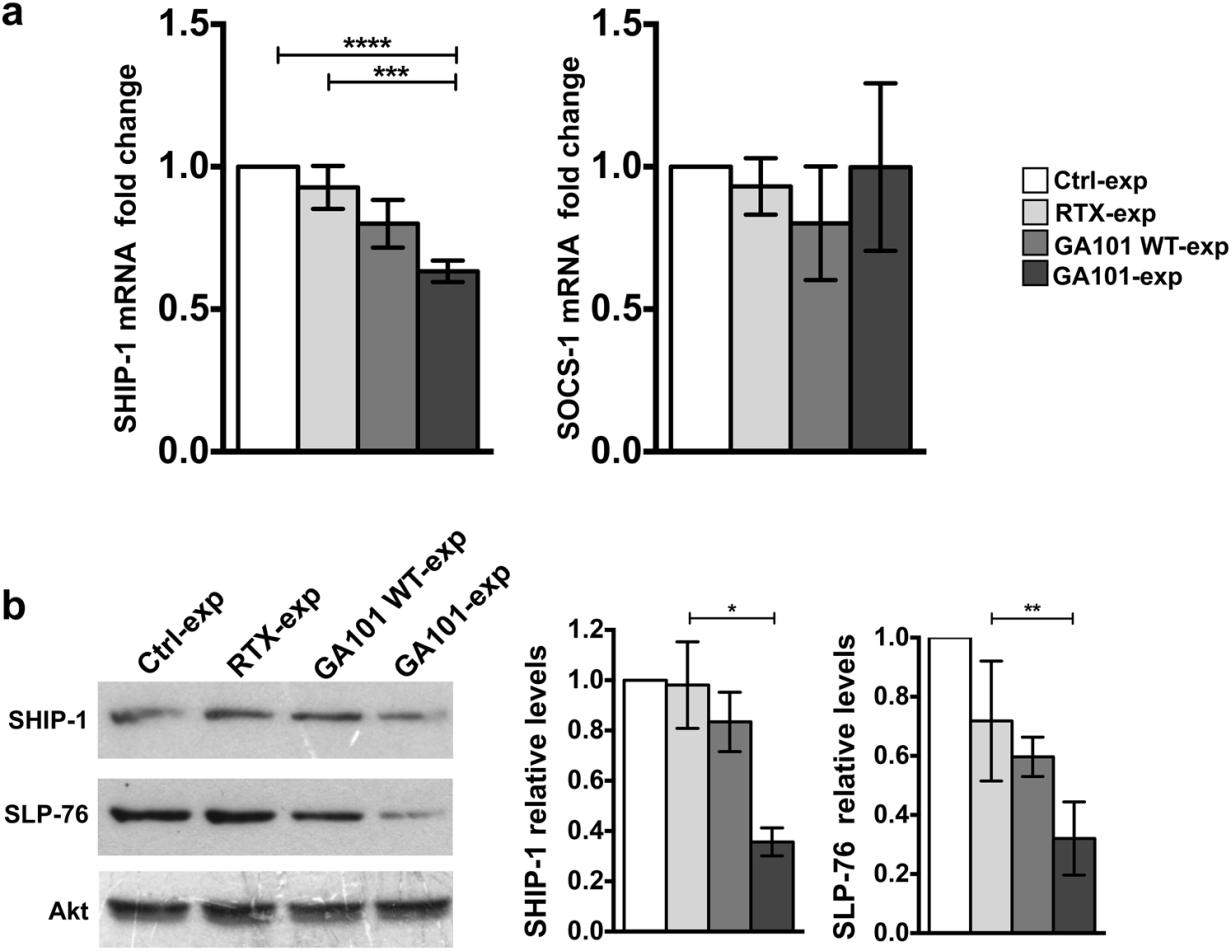
SHIP-1 and SLP-76 downregulation in obinutuzumab-experienced NK cells. Primary cultured NK cells were stimulated (2:1) for 18 h with biotinylated rituximab (RTX-exp)-, obinutuzumab WT (GA101 WT-exp)-, obinutuzumab (GA101-exp)-opsonized or not opsonized Raji (Ctrl-exp) and immunomagnetically purified by negative selection. **a** Relative SHIP-1 or SOCS-1 mRNA levels were measured by RT-qPCR. The fold change expression relative to Ctrl-exp population (set to 1) after normalizing with the GAPDH endogenous control is reported in the bar graph. Data (mean ± SEM) from *n* = 15 donors of three independent experiments are shown. ****p < 0.0001, ***p = 0.0001. **b** Western blot analysis of equal amounts of total lysates. The same membrane was immunoblotted as indicated. One representative experiment is shown (left panels). The relative values of SHIP-1 or SLP-76 were obtained by normalizing to the levels of Akt and are expressed as fold change respect to control population (set to 1). Data (mean ± SEM, *n* = 8) from three independent experiments are depicted in bar graphs, **p = 0.0078, *p = 0.0156 (right panels)

Taking into account that SHIP-1 and SOCS-1 signaling intermediates, both relevant to IFN-γ production in NK cells, are known to be direct targets of miR-155 [13, 14, 32, 33], we assessed their expression levels in anti-CD20-experienced cells. We observed a significant reduction of SHIP-1 mRNA in obinutuzumab-, but not in rituximab- or GA101WT-experienced cells, with respect to control population (Fig. 4a, left). On the contrary, SOCS-1 mRNA levels were not modulated at significant levels (Fig. 4a, right).

Accordingly, we found a marked reduction of SHIP-1 at protein level of almost 65% in obinutuzumab-experienced cells, but not in rituximab- or GA101WT-stimulated samples, with respect to control population. A consensual downregulation of the signaling intermediate SLP-76, known to be a direct target of miR-155 too [32], was observed in obinutuzumab-experienced cells (Fig. 4b).

Collectively, these data demonstrate that the sustained CD16 ligation by means of obinutuzumab-opsonized targets induces the upregulation of miR-155 levels associated with the downregulation of both SHIP-1 and SLP-76.

### Role of PI3K/mTOR pathway in the increased IFN-γ competence of obinutuzumab-experienced NK cells

The acute reduction of SHIP-1 in NK cells may lead to the amplification of PI3K-dependent signals, which is responsible for an increased IFN-γ production [35, 36].

**Fig. 5:**
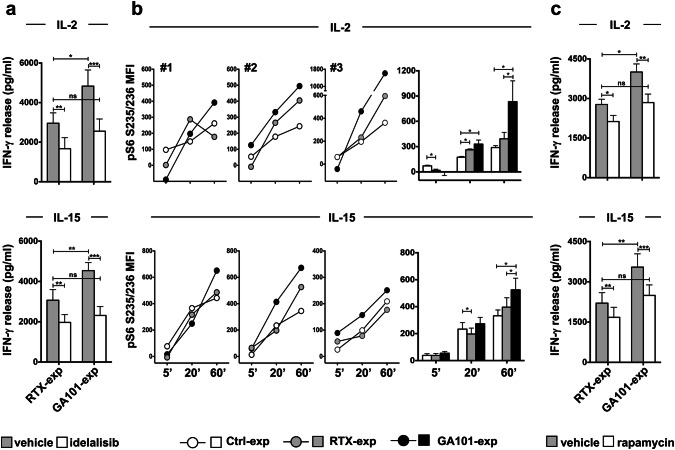
Amplified γ_c_ cytokine-dependent S6 phosphorylation and increased sensitivity to PI3K/mTOR inhibition in obinutuzumab-experienced NK cells. Primary cultured NK cells were stimulated (2:1) for 18 h with biotinylated rituximab (RTX-exp)-, obinutuzumab (GA101-exp)-opsonized (**a**–**c**) or not opsonized Raji (Ctrl-exp) (**b**) and immunomagnetically purified by negative selection. Experienced NK cells were treated with 10 μM idelalisib (**a**), 20 nM rapamycin (**c**) (white histograms) or with the same volume of DMSO as vehicle (grey histograms) for 2 h and then stimulated for additional 18 h with IL-2 (500 U/ml) or IL-15 (100 ng/ml), as indicated, in the presence of the inhibitors. Supernatants were then collected and assessed for IFN-γ levels. Data (mean ± SEM) from *n* = 8 donors from three independent experiments are depicted in bar graphs. ***p < 0.0008, **p < 0.0006, *p < 0.03. **b** Experienced NK cells were stimulated with IL-2 (500 U/ml) (top panels) or IL-15 (100 ng/ml) (bottom panels) for the indicated times. The phosphorylation levels of ribosomal protein S6 in S235/236 residues were evaluated by FACS analysis in fixed and permeabilized samples. Graphs depict for each time point the MFI value calculated as follow: (MFI of cytokine activated sample-MFI of untreated sample). Three representative donors and mean ± SEM from *n* = 6 donors are shown. *p = 0.031

To assess the functional role of PI3K in the priming of obinutuzumab-experienced cells, IL-2- or IL-15-dependent IFN-γ production was assessed in anti-CD20-experienced NK cells in the presence or absence of the p110δ PI3K inhibitor, idelalisib. As expected, the production of IFN-γ was reduced in the presence of inhibitor, although with a different sensitivity between populations; in fact, idelalisib completely counteracts the enhancement of IFN-γ production observed in obinutuzumab-experienced cells (Fig. 5a).

Downstream of PI3K, mTOR acts in promoting mRNA translation [15]. We assessed ribosomal protein S6 phosphorylation in response to cytokine stimulation. The basal phosphorylation levels are comparable between all the experimental groups (Supplementary Fig. 2). In control population, the stimulation with IL-2 or IL-15 induces a time-dependent increase of S6 phosphorylation up to 60 min (Supplementary Fig. 3a). In obinutuzumab-experienced cells, in response to γ_c_ cytokines, we observed higher levels of S6 phosphorylation, particularly at later time points of stimulation, with respect to rituximab- or not opsonized target-experienced cells, which behave comparably (Fig. 5b).

The functional role of the mTOR/S6K pathway was evaluated by assessing IFN-γ production in the presence of the mTOR inhibitor, rapamycin. Rapamycin treatment inhibits, in a dose-dependent manner, cytokine-induced S6 phosphorylation (Supplementary Fig. 3b); by comparing the effect of rapamycin on anti-CD20-experienced populations, we observed a more pronounced inhibition of IFN-γ production in obinutuzumab- with respect to rituximab-experienced cells (Δ between untreated and treated cells is 1158 for GA101-exp vs 647 for RTX-exp for IL-2 setting; 1051 for GA101-exp vs 530 for RTX-exp for IL-15 setting) (Fig. 5c). Indeed, rapamycin prevents the enhanced response of obinutuzumab-experienced cells.

All together, these data indicate that the enhanced IFN-γ production in response to IL-2 or IL-15 stimulation relies on a potentiated PI3K/mTOR/S6K axis.

## Discussion

**Fig. 6:**
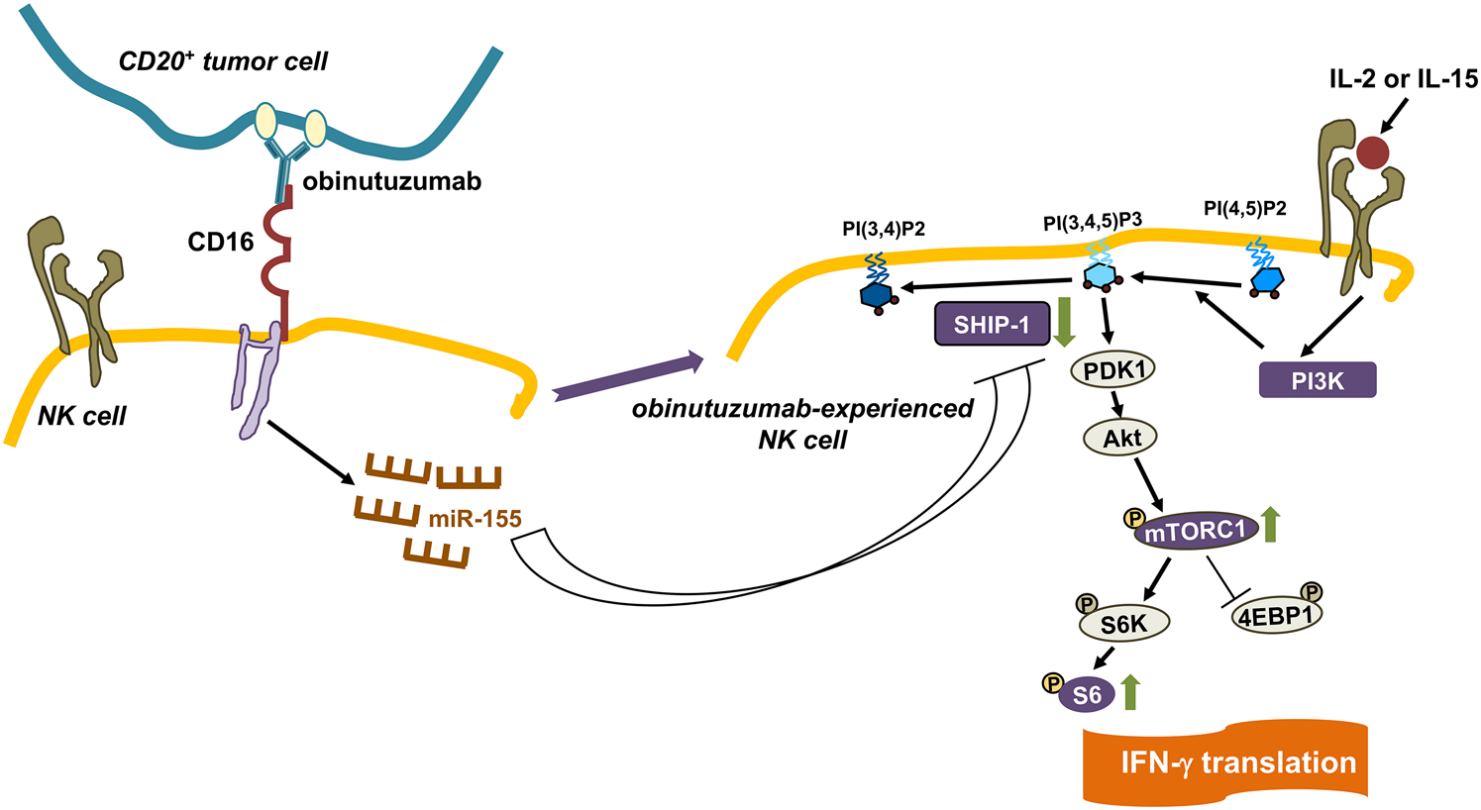
Obinutuzumab-experienced NK cells exhibit γ_c_ cytokine hyperresponsiveness. CD16-dependent miR-155 promotes SHIP-1 downregulation which in turn potentiates the PI3K/mTOR pathway responsible for enhanced IFN-γ translation

We show here that the outcome of a sustained (18 h) interaction of NK cells with obinutuzumab-opsonized targets is an increased capability to produce IFN-γ in response to high doses of IL-2 or IL-15 which depends on a potentiated PI3K/mTOR pathway associated with miR-155 upregulation and SHIP-1 downregulation (Fig. 6) [1].

Recently, Pahl et al. demonstrated that CD16 aggregation by a tetravalent bispecific therapeutic antibody AFM13 (CD30/CD16A) followed by long-term re-stimulation with IL-2 or IL-15 results in a global NK cell hyperresponsiveness, attributable to increased cytokine receptor expression levels [37]. In our experimental conditions, we observed that the improved sensitivity of obinutuzumab-experienced cells to cytokine receptor stimulation coincided with increased CD25 expression levels, which, together with CD122 (IL-2Rβ) and CD132 (γ_c_), assembles the trimeric high-affinity IL-2R [38], whereas IL-15R α chain, which by itself mediates the high-affinity binding [27, 39], remains unaffected upon CD16 stimulation. Such observations, along with the fully stimulatory doses of cytokines used, would exclude an increased receptor affinity as the unique mechanism responsible for the enhanced responsiveness.

The lack of correlation between IFN-γ protein and mRNA levels in γ_c_ cytokine-stimulated samples led us to hypothesize that post-transcriptional mechanisms may be responsible for the amplified response. In this context, our data confirm the existence of fundamental differences in the regulation of IFN-γ production induced by different cytokines [12, 40, 41]; in response to stimulation with IL-12, IFN-γ transcript increases almost 20-fold, whereas stimulation with IL-2 or IL-15 leads to a lower IFNG gene transcription, despite the efficient IFN-γ production. The stronger transcriptional effect of IL-12 is in line with a necessary role of STAT4 for maximal IL-12-mediated IFN-γ transcription [40, 41]. The increased IFN-γ competence is reminiscent of the recently described memory NK cell population which, in fact, exhibits enhanced IFN-γ production in response to CD16 stimulation [26]; however, memory NK cells display a defective responsiveness to IL-12 stimulation [42].

Several recent studies have demonstrated that IFN-γ production by NK cells is under the control of the metabolic sensor mTOR [16, 43, 44]. PI3K/Akt/mTOR pathway is an essential component of signaling via IL-15R and IL-2R [16, 45], with mTOR involved in translational activation through the phosphorylation of 4E-BP1 and S6K [15, 17, 18, 45].

In this context, our data evidence, in obinutuzumab-experienced cells, an amplified ribosomal protein S6 phosphorylation induced by γ_c_ cytokine stimulation along with a higher sensitivity to the mTOR inhibitor rapamycin, thus indicating that the increased IFN-γ competence depends on the mTOR pathway.

Based on these data, we reasoned that CD16 ligation in high-affinity conditions may lower the threshold for PI3K/mTOR pathway activation, thus leading to γ_c_ cytokine hyperresponsiveness.

SHIP-1 phosphatase acts in constraining PI3K/Akt-dependent signals [46] and negatively regulates IFN-γ production by monokines and CD16 stimulation in both human and mouse NK cells [35, 36]; its reduced expression levels in CD56bright with respect to CD56dim NK cells contribute to the increased IFN-γ producing potential of the former population [34]. SHIP-1, along with SOCS-1, are directly targeted and repressed by miR-155, which is involved in the regulation of IFN-γ production stimulated by cytokines in NK cells [13, 14, 32, 33].

On this line, we evidence that the upregulation of miR-155 transcript, in obinutuzumab-experienced cells, associates with SHIP-1 but not SOCS-1 downregulation; the observation that both rituximab and obinutuzumab WT stimulation induce still significant but lower levels of miR-155 indicates that the strength of CD16 ligation dictates the capability to promote miR-155 expression. On this issue, a recent paper highlighted a correlation between the strength of TCR stimulation and miR-155 expression levels that impacts IFN-γ production by anti-tumor CD8^+^ T cells [47].

SLP-76, a direct target of miR-155 in NK cells [32], is known to orchestrate the formation of a molecular platform critical in signal integration downstream activating receptors [2]. The reduction of SLP-76 levels, along with the degradation of FcεRIγ and CD3ξ adaptor chains and the Spleen-associated tyrosine kinase, Syk [25], may explain the profound impairment of IFN-γ production induced by the engagement of NKG2D and 2B4 activating receptors in anti-CD20-experienced cells. It should be noted that in a different experimental setting, based on shorter CD16 pre-ligation (90 min), we previously reported that the capability to produce IFN-γ in response to activating receptor stimulation was preserved [24]. In such conditions, no miR-155 upregulation and consequently no SLP-76 downmodulation have occurred (C. Capuano, unpublished observation).

The role of PI3K in the priming of obinutuzumab-experienced cells is strengthened by the observation that the inhibition of PI3K greatly counteracts γ_c_ cytokine hyperresponsiveness, while, as previously described, it has a more marginal effect on NK cell viability and cytotoxicity [48].

NK cell plasticity is being exploited by new NK cell-based intervention strategies against cancer [49–51]; by recapitulating the signaling pathway responsible for CD16-dependent priming, this study evidences new aspects of NK cell adaptation in a therapeutic setting. Taking into account that NK-derived IFN-γ is a key immunoregulatory factor in the shaping of anti-tumor adaptive immune responses, obinutuzumab-based therapy may be envisaged as a driver of a mAb-mediated vaccinal effect [5–9] that could significantly impact on long-term therapeutic efficacy. Suitable clinical trials focused on the prospective evaluation of the emergence of adaptive anti-tumor responses in obinutuzumab-treated patients are needed to highlight the actual translational impact.

### Electronic supplementary material

Below is the link to the electronic supplementary material.

Supplementary file1 (PDF 1528 kb)

## Abbreviations

4E-BP: eukaryotic translation initiation factor 4E (eIF4E)-binding protein 1
γ_c_: common γ chain
EGTA: ethylene glycol tetraacetic acid
Fc: crystallizable fragment
GAPDH: elyceraldehyde-3-phosphate dehydrogenase
IL-2R: IL-2 receptor
IL-15R: IL-15 receptor
miR: microRNA
mTOR: mammalian target of rapamycin
NKG2D: Natural Killer group 2 member D
PIP3: phosphatidylinositol 3,4,5-trisphosphate
RT-qPCR: real-time quantitative PCR
S6K: ribosomal protein S6 kinase
SLP-76: src homology 2 domain-containing leukocyte protein of 76 kDa
SOCS-1: suppressor of cytokine signaling 1
WT: wild type
