# Supplementary material for: CD16 pre-ligation by defucosylated tumor-targeting mAb sensitizes human NK cells to γc cytokine stimulation via PI3K/mTOR axis
Source: Cancer Immunol Immunother. 2020 Jan 16;69(4):501–12. doi: 10.1007/s00262-020-02482-2 (PMC7113231; doi:10.1007/s00262-020-02482-2)
Supplement: Supplementary file 1 — Supplementary file1 (PDF 1528 kb) [file 262_2020_2482_MOESM1_ESM.pdf]

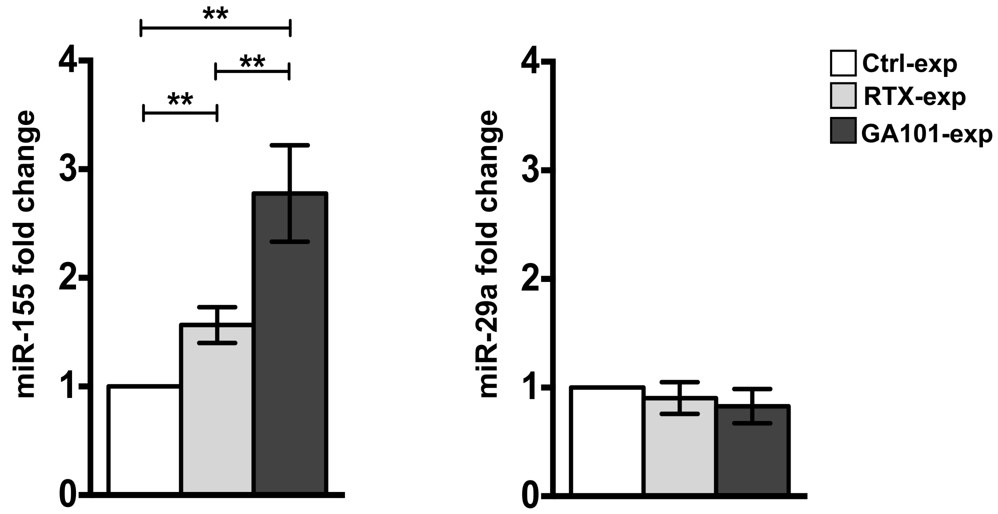

supplementary Figure 1. miR-155 upregulation in anti-CD20-experienced NK cells**.**

Primary cultured NK cells were stimulated (2:1) for 18 h with biotinylated rituximab (RTX-exp)-, obinutuzumab (GA101-exp)-opsonized or not opsonized Raji (Ctrl-exp) and immunomagnetically purified by negative selection. Relative miR-155 or miR-29a levels were measured by RT-qPCR. Bar graphs depict the fold change expression relative to the Ctrl-exp population (set to 1) after normalizing with the RNU-48 endogenous control. Data (mean ± SEM) from n=9 donors from three independent experiments are shown. ***p*=0.0039

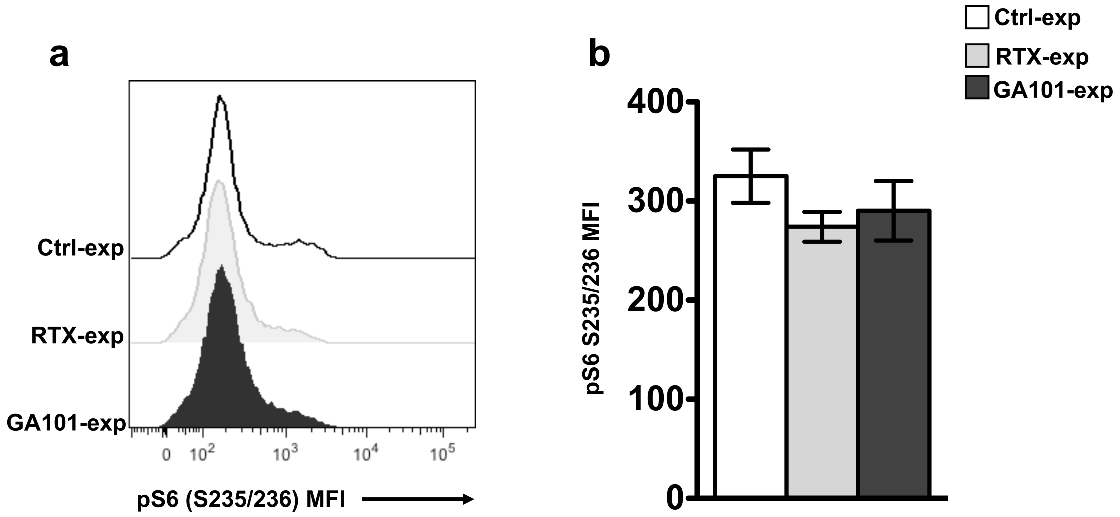

supplementary Figure 2 Basal S6 phosphorylation levels in experienced NK cells.

Primary cultured NK cells were stimulated (2:1) for 18 h with biotinylated rituximab (RTX-exp)-, obinutuzumab (GA101-exp)-opsonized or not opsonized Raji (Ctrl-exp) and immunomagnetically purified by negative selection. S6 phosphorylation levels in S235/236 residues were evaluated by FACS analysis in fixed and permeabilized samples. **a** Histogram overlay from one representative experiment is shown. **b** MFI values (mean ± SEM) from n=8 donors from three independent experiments

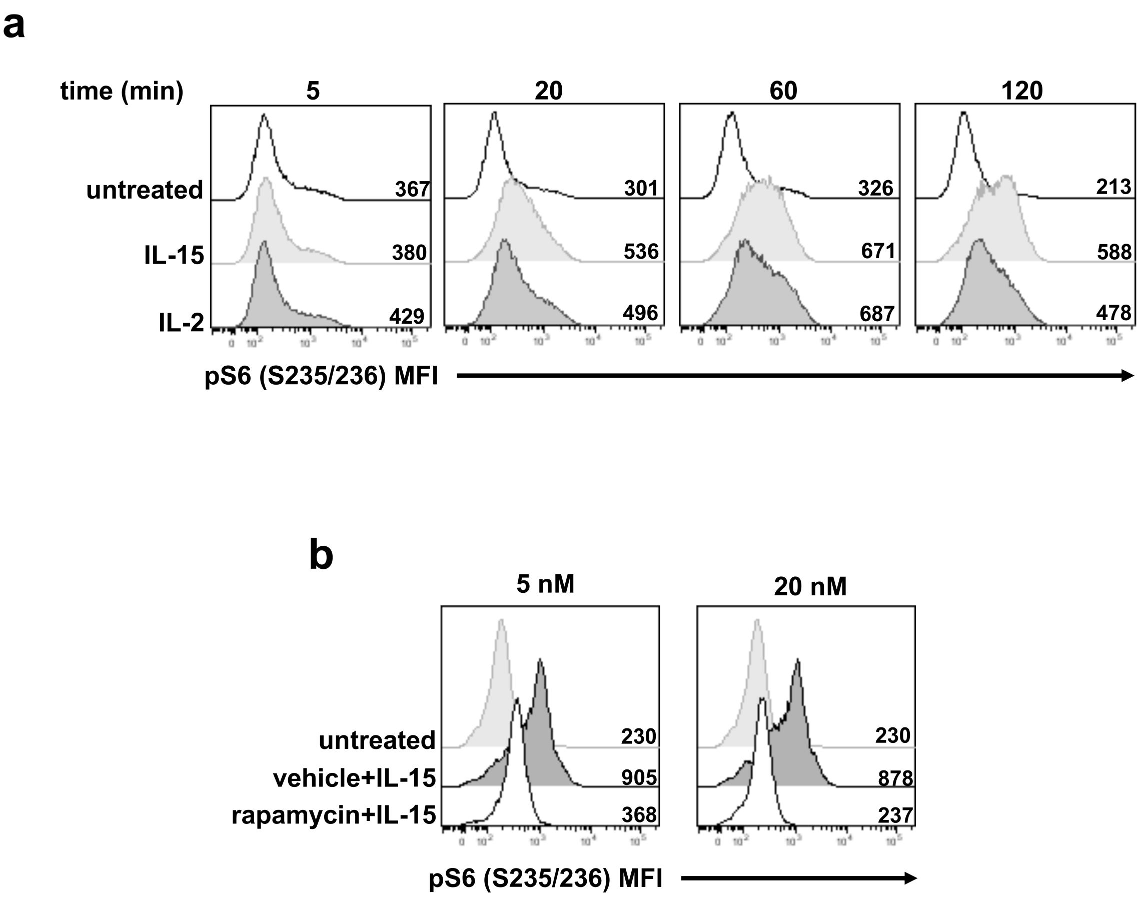

supplementary Figure 3 Kinetics of γ_c cytokine-induced S6 phosphorylation and sensitivity to mTOR inhibitor rapamycin.

Primary cultured NK cells were stimulated (2:1) for 18 h with biotinylated not opsonized Raji and immunomagnetically purified by negative selection. **a** Cells were left unstimulated or activated with IL-2 (500 U/ml) or IL-15 (100 ng/ml) for the indicated times. **b** NK cells were left untreated (light grey) or pretreated with the indicated doses of rapamycin (white) or with the same volume of DMSO as vehicle (dark grey) for 2 h, and then stimulated for additional 60 min with IL-15 (100 ng/ml) in the presence of the inhibitor. **a** and **b** The phosphorylation levels of ribosomal protein S6 in S235/236 residues were evaluated by FACS analysis in fixed and permeabilized samples. Histogram overlays from one representative experiment, out of three performed, are shown. Numbers represent the MFI values.

supplementary Table 1. CLL patient characteristics

Patient	Age	Sex	%CD5/CD19	%CD56/CD16	RAI stage
1	68	male	73.0	9.5	0
2	68	male	96.8	1.33	2
3	52	female	86.2	4.47	0
